# Phytochemical Analysis and *In Vitro* Antileukemic Activity of Alkaloid-Enriched Extracts from *Vinca sardoa* (Stearn) Pignatti

**DOI:** 10.3390/molecules28155639

**Published:** 2023-07-25

**Authors:** Daniela De Vita, Claudio Frezza, Fabio Sciubba, Chiara Toniolo, Camilla Badiali, Rita Petrucci, Martina Bortolami, Paola Di Matteo, Daniele Rocco, Annarita Stringaro, Marisa Colone, Andrea Maxia, Maria Teresa Petrucci, Mauro Serafini, Sebastiano Foddai

**Affiliations:** 1 Department of Environmental Biology, “Sapienza” University of Rome, Piazzale Aldo Moro 5, 00185 Rome, Italy; claudio.frezza@uniroma1.it (C.F.); fabio.sciubba@uniroma1.it (F.S.); chiara.toniolo@uniroma1.it (C.T.); camilla.badiali@uniroma1.it (C.B.); mauro.serafini@uniroma1.it (M.S.); sebastiano.foddai@uniroma1.it (S.F.); 2 NMR-Based Metabolomics Laboratory, “Sapienza” University of Rome, Piazzale Aldo Moro 5, 00185 Rome, Italy; 3 Department of Basic and Applied Sciences for Engineering, “Sapienza” University of Rome, Via Castro Laurenziano 7, 00161 Rome, Italy; rita.petrucci@uniroma1.it (R.P.); martina.bortolami@uniroma1.it (M.B.); p.dimatteo@uniroma1.it (P.D.M.); daniele.rocco@uniroma1.it (D.R.); 4 National Center for Drug Research and Evaluation, Italian National Institute of Health, Viale Regina Elena 299, 00161 Rome, Italy; annarita.stringaro@iss.it (A.S.); marisa.colone@iss.it (M.C.); 5 Laboratory of Economic and Pharmaceutical Botany, Department of Life and Environmental Sciences, University of Cagliari, Viale S. Ignazio da Laconi 13, 09123 Cagliari, Italy; a.maxia@unica.it; 6 Department of Cellular Biotechnologies and Hematology, “Sapienza” University of Rome, Via Benevento 6, 00161 Rome, Italy; petrucci@bce.uniroma1.it

**Keywords:** *Vinca sardoa* (Stearn) Pignatti, *Vinca difformis* subsp. *sardoa* Stearn, Sardinian periwinkle, alkaloids, antileukemic activity, ESI-MS/MS characterization, HPLC-PDA-ESI-MS analysis

## Abstract

*Vinca sardoa* (Stearn) Pignatti, known as Sardinian periwinkle, is widely diffused in Sardinia (Italy). This species contains indole alkaloids, which are known to have a great variety of biological activities. This study investigated the antileukemic activity against a B lymphoblast cell line (SUP-B15) of *V. sardoa* alkaloid-rich extracts obtained from plants grown in Italy, in Iglesias (Sardinia) and Rome (Latium). All the extracts showed a good capacity to induce reductions in cell proliferation of up to 50% at the tested concentrations (1–15 µg/mL). Moreover, none of the extracts showed cytotoxicity on normal cells at all the studied concentrations.

## 1. Introduction

**Figure 1:**
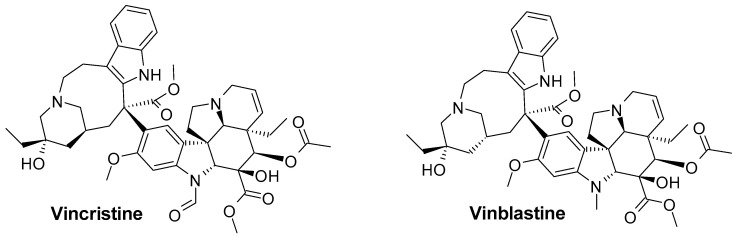
Chemical structure of vincristine and vinblastine.

*Vinca sardoa* (Stearn) Pignatti syn. *Vinca difformis* subsp. *sardoa* Stearn (Apocynaceae) is a perennial herbaceous widely diffused in Sardinia (Italy) where it is called Sardinian periwinkle [1]. From the morphological point of view, *V. sardoa* is very similar to the species *Vinca difformis* Pourret with the exception of the leaves: *V. difformis* has ovate and glabrous leaves, while *V. sardoa* has ovate-acuminate leaves with hairs up to 0.2 mm on the leaf margin [2]. The two species differ in the phytochemical analysis of the alkaloids, as we described previously [3]. The roots and leaves of *V. sardoa* contain indole alkaloids e.g., norfluorocurarine, akuammigine, conoflorine, and venalstonine [3,4,5]. Indole alkaloids are widely distributed in Angiosperm, including Apocynaceae, Rubiaceae, and Loganiaceae [6], as well as in the fungi kingdom [7]. The use of indole alkaloids has a long history, both in shamanic ceremonies [7] and in the pharmaceutical field [8]. Currently, indole alkaloids are known to show a variety of biological activities such as antimicrobial [9], anti-depressant [8,10], antiviral [11], and anticancer ones [12,13]. Some of them, vincristine and vinblastine (Figure 1), are even used for the treatment of some kind of cancers [14] such as lymphomas and acute lymphoblastic leukaemia (ALL) [15].

ALL is a paediatric malignancy that rarely affects adults. Patients affected by ALL show proliferation and accumulation of malignant and immature lymphatic blasts in the bone marrow, peripheral blood, and lymphatic and non-lymphatic tissue. If untreated, the disease is fatal within a few months from the diagnosis [16]. Despite the available drugs, the side effects associated with chemotherapy, mainly neurotoxicity, cardiotoxicity [17], and hepatotoxicity [18], together with drug resistance make new therapeutic options necessary [19].

Due to the presence of indole alkaloids in *V. sardoa*, we decided to investigate the *in vitro* activity of the alkaloid-enriched extract from *V. sardoa* on a B lymphoblast cell line (SUP-B15) and a normal cell line (fibroblast), by studying the effects on cell viability.

## 2. Results and Discussion

### 2.1. Extraction of V. sardoa Aerial Parts

For this study, we considered the aerial parts of *V. sardoa* collected in Iglesias (Italy) in 2019 and 2021 and in the experimental botanical garden of Sapienza University of Rome in 2019. The three samples were treated with a diluted solution of acetic acid. The acidic solutions were made alkaline with sodium bicarbonate and finally extracted with dichloromethane. The organic extracts gave a positive result to Dragendorff’s reagent indicating the presence of alkaloids. The extracts obtained from the plants collected in Iglesias in 2019 and 2021 were named IG2019 and IG2021, respectively, while the extract obtained from the plant collected in Rome was named RM2021. All the extracts were analysed by LC-MS and NMR spectroscopy, as discussed below.

### 2.2. LC-PDA-ESI-MS and DI-ESI-MS/MS Analysis of the Extracts

**Figure 2:**
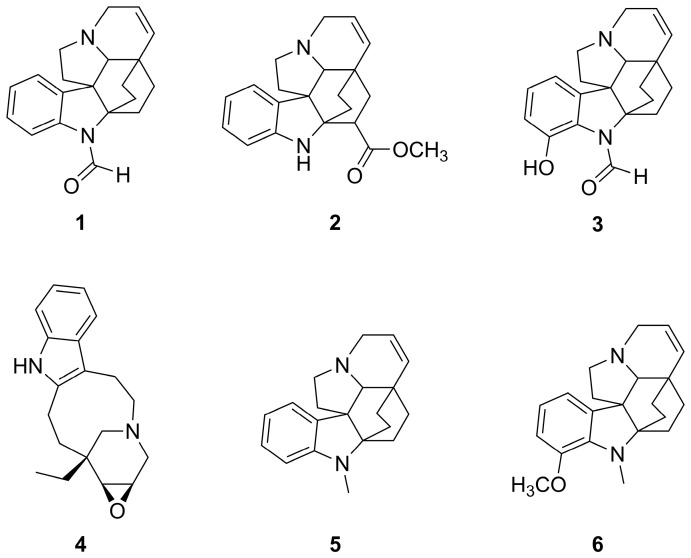
Structures of alkaloids **1**–**6** detected in the extracts of *Vinca sardoa* (Stearn) Pignatti by HPLC-PDA-ESI-MS analysis.

The previously obtained [5] alkaloids **1**–**6** (Figure 2) were analysed individually by direct infusion (DI) into the electrospray ionization (ESI) source, in tandem mass spectrometry (MS/MS) experiments.

Although they had been previously characterized [5], the ESI-MS/MS fragmentation spectra are herein provided for each compound for the first time (Figures S1–S6).

All the compounds except **4** exhibited two characteristic fragmentations: (a) the loss of 17 mass units, containing an N atom since an odd mass fragment is obtained, that has been ascribed to the loss of NH_3_ (Figures S1–S3, S5 and S6); (b) the loss of 135 mass units (Figures S1, S3, S5 and S6), that becomes 134 + 59 = 193 in the case of **2** (Figure S2).

All compounds gave a characteristic fragment that has been assigned to a positive indole-like fragment with 144 *m*/*z* (Figures S1, S2 and S4–S6) or to corresponding hydroxyl-substituted fragment with 160 *m*/*z* (Figure S3).

**Figure 3:**
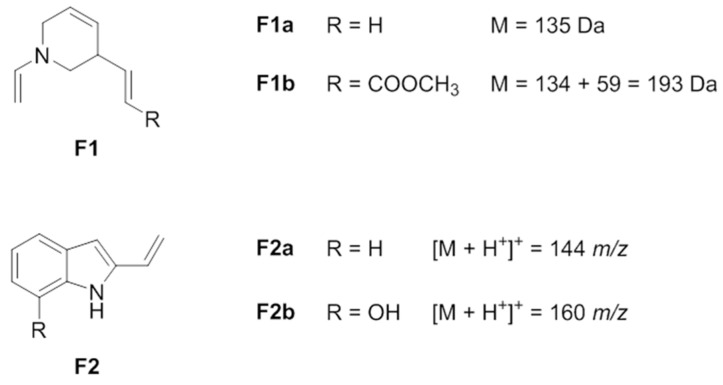
Suggested structures of characteristic lost fragments and positive fragments of alkaloids **1**–**6** by DI-ESI-MS/MS experiments.

The structures of the tentatively assigned lost fragments, corresponding to −135 and −193 mass units, are shown in Figure 3 (F1a and F1b, respectively); the structures of the tentatively assigned positive fragments with 144 *m*/*z* and 160 *m*/*z* are shown in Figure 3 (F2a and F2b, respectively).

Moreover, similar fragmentation patterns were observed for structurally similar alkaloids.

Briefly, *N*(1)-formyl-14,15-didehydroaspidofractinine (**1**) and *N*(1)-formyl-14,15-didehydro-12-hydroxyaspidofractinine (**3**) exhibit the same fragmentation pattern, with fragments differing by 16 mass units, according to the presence (**3**) or not (**1**) of the –OH moiety; they are characterized by a base peak at [M + H^+^-45]^+^, at 262 and 278 *m*/*z*, respectively, likely due to the loss of the *N*-formyl moiety (-H_2_NCHO); a second peak likely arises from the loss of 135 (described above) combined with the loss of CO (−28) to give the positive fragments 144 *m*/*z* and 160 *m*/*z* (Δ = 16, as described above), respectively (**1** and **3**, in Figures S1 and S3, respectively).

*N*(1)-methyl-14,15-didehydroaspidofractinine (**5**) and *N*(1)-methyl-14,15-didehydro-12-methoxyaspidofractinine (**6**) exhibit the same fragmentation pattern, with fragments differing by 30 mass units, according to the presence (**6**) or not (**5**) of the –OCH_3_ moiety; they are characterized by the base peak corresponding to the ion [M + H^+^-135]^+^ at 158 and 188 *m*/*z*, respectively. The loss of −14 mass units corresponds to the loss of the *N*(1)-methyl moiety as –CH_2_ gives the fragment 144 *m*/*z* for compound **5** and the fragment 174 *m*/*z* for compound **6**, the last one giving the fragment 144 *m*/*z* by further loss of the methoxy moiety (−30 mass units). Moreover, **5** and **6** fragmentation patterns evidenced a characteristic ion [M + H^+^-120]^+^ at 173 and 203 *m*/*z*, respectively, not assigned but whose even neutral mass value (172 and 202 Da, respectively) indicates the presence of both N atoms in the fragment (**5** and **6**, in Figures S5 and S6, respectively).

Compound **2**, venalstonine (PubChem NSC180520), exhibited the ion [M + H^+^-NH_3_]^+^ = 320 *m*/*z* as peak base; due to the -COOCH_3_ moiety, the characteristic loss of 135 discussed above appears as the loss of 193 mass units (see Figure 3, F1b). Another fragment at 260 *m*/*z* was likely due to the loss of the same group as HCOOCH_3_, as shown in Figure S2.

Compound **4**, conoflorine, exhibits a fragmentation pattern slightly differing from the others. From the epoxide moiety arises a small peak for the typical fragment [M + H^+^-18]^+^ = 279 *m*/*z* due to the loss of water. Also in this case, the pattern evidenced the indole-like fragment (F2a, in Figure 3) at 144 *m*/*z*; the non-indole residual fragment at 154 *m*/*z* is the base peak, confirmed by the fragment at 136 *m*/*z* corresponding to the loss of water due to the epoxide moiety (**4**, in Figure S4).

The strong correlation observed between the analysed alkaloid structure and the fragmentation profile provides a powerful tool for the characterization of other indole alkaloids in the future.

The purified samples of alkaloids **1**–**6** were used to optimize the chromatographic separation and the mass spectral parameters for the selected ion recording (SIR) mode analysis, used for their selective and sensitive unambiguous identification in the IG2019, IG2021, and RM2019 extracts.

The three different samples of *V. sardoa* exhibit a similar profile: in fact, alkaloids **1**–**6** were identified in all samples, by comparison of chromatographic and mass spectral data with the purified samples, as reported in Table 1. The typical chromatographic profile of the extracts is shown in Figure S7a, in which the total ion chromatogram (TIC) of the RM2019 extract is shown as an example. The selected *m*/*z* mass values 307, 337, 323, 297, and 293 corresponding to the alkaloids **1**, **2**, the isobaric **3** and **6**, **4**, and **5**, respectively, are evidenced in the corresponding SIR chromatograms (Figure S7b–f).

On the basis of the total ions detected in each SIR channel, alkaloid **5** seemed the most abundant one (Figure S7f, TIC = 4.00 × 10^8^), followed by alkaloid **4** (Figure S7e, TIC = 2.60 × 10^8^), while alkaloid **1** was in general the least abundant one (Figure S7b, TIC = 10.00 × 10^7^), in good agreement with NMR data described below.

### 2.3. NMR Analysis

Selected samples of *V. sardoa* extracts, namely RM2019, IG2019, and IG2021, were analysed by ^1^H-NMR spectroscopy to quantify the identified alkaloids. The spectra showed only quantitative differences among them, but no qualitative ones, so a representative spectrum is reported in Figure S8. The spectra showed resonances attributable to sterols, fatty acids, and terpenes according to the literature data [20], as well as the resonances of the alkaloids identified by LC-MS. The resonances of the quantified alkaloids, in particular the ones of the unsaturated protons, were attributed on the basis of the literature data (PII: S0031-9422(97)00533-S) and were quantified by integration of their diagnostic aromatic resonances. In greater detail, the molecules **1**, **2**, and **5** were identified by their diagnostic unsubstituted aromatic ring (doublet at 6.40 ppm, triplet at 6.71 ppm, triplet at 6.83 ppm, and doublet at 7.31 ppm) and quantified by the resonance at 7.31 ppm; indole **4** was identified on the basis of its indole-like resonances (triplet at 7.29 ppm, triplet at 7.38 ppm, doublet at 7.55 ppm, and doublet at 8.14 ppm) and quantified by the resonance at 7.55 ppm. Compounds **3** and **6** were identified on the basis of the substituted 6-terms aromatic ring (doublet at 6.71 ppm, doublet at 6.81 ppm, and triplet at 7.08 ppm) and quantified by the resonance at 6.81 ppm. The integrals related to molecule signals were normalized by one of the internal standards (normalized by the number of protons) and then converted into mg/100 g of dried extract. Since the superimposition of several molecules with similar structures, the amounts of **1**, **2**, and **5** are reported as equivalents of molecule **1**, while compounds **3** and **6** are reported in Table 2.

### 2.4. Cytotoxicity Assays

**Figure 4:**
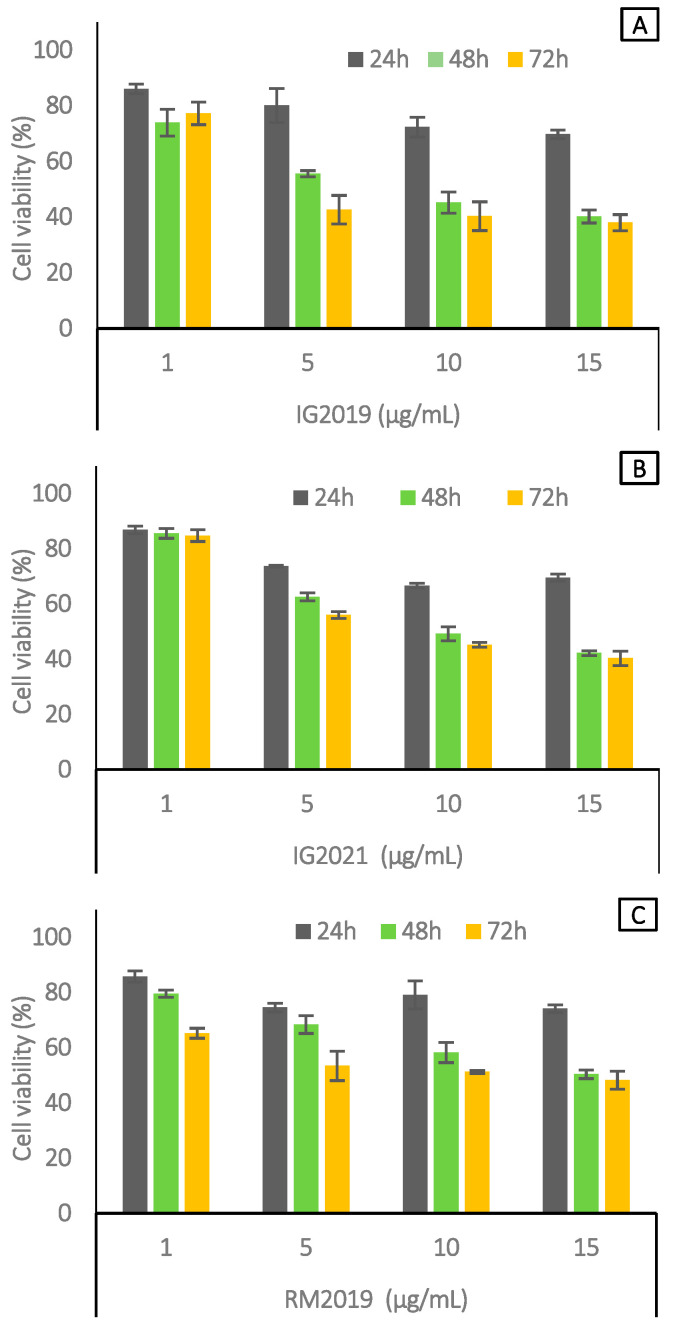
Cell viability analysis in SUP-B15 cells treated with IG2019 (**A**), IG2021 (**B**), and RM2019 (**C**) at 24, 48, and 72 h. All experiments were performed in triplicate and the data are expressed as mean ± SD values.

Cell cytotoxic activity of the extract of *V. sardoa* (Figure 4) on lymphoblast cell line SUP-B15 was evaluated by MTT assay at different times (24, 48, and 72 h) with different concentrations (1 µg/mL, 5 µg/mL, 10 µg/mL, and 15 µg/mL, respectively). The results suggested that, after 48 h of the treatment, the concentration of 5 µg/mL for the IG2019 extract was able to induce a reduction in cell proliferative capacity (40–45%). Moreover, after 48 and 72 h, higher concentrations (10 and 15 µg/mL) of the IG2019 extract induced a proliferation percentage decrease (40%) (Figure 4A). In addition, the IG2021 extract had the same effect as the IG2019 extract from 10 to 15 µg/mL at 48 and 72 h after treatments (Figure 4B). RM2019 showed a reduction in cell viability lower than 45% (Figure 4C) at 72 h at the concentration of 15 µg/mL. Furthermore, we evaluated the cytotoxic activity of plant extracts on normal cells such as human fibroblasts. Figure S9 shows that none of the extracts exhibited cytotoxic activity at all concentrations and times used, with the exception of IG2019, which induced cytotoxicity at higher concentration (15 µg/mL) after 72 h. Vincristine and vinblastine were used as reference drugs. The cytotoxicity assay results are reported in Figure S10.

## 3. Materials and Methods

### 3.1. Plant Material

The aerial parts (stem and leaves) of *V. sardoa* were collected in October 2019 and October 2021 in the area of Iglesias, Sardinia, Italy (geographical coordinates 41°46′16″ N, 13°21′39″ E); the botanical identification was carried out by one of us (A.M.). Representative samples of this collection are stored in the Pharmaceutical Biology laboratory (Department of Environmental Biology, Sapienza University of Rome) for further reference, under the voucher names IG2019 and IG2021, respectively. Moreover, a third sample (RM2019) was collected in late September 2019 from a living plant hosted at the Experimental Garden of Sapienza University of Rome.

### 3.2. Chemicals

Analytical grade chemicals were provided by Sigma-Aldrich (Milan, Italy) and used without further purification. The solvents were HPLC-grade and were provided by Carlo Erba (Milano, Italy), while deionized water was prepared daily using a Milli-Q purification system (Millipore, Vimodrone, Italy).

### 3.3. Extraction Procedure

The extracts were obtained by a 48 h maceration of the coarsely minced aerial parts of *V. sardoa* in 2% aq. AcOH (plant material (g)/solvent (mL) ratio of 1:35). The aqueous solution was made alkaline with Na_2_CO_3_ until pH ~8–9 and extracted with dichloromethane (3×). The reunited organic phases were dried over Na_2_SO_4_ and the organic solvent was evaporated by a rotary evaporator equipped with a water bath heated to 30 °C. A brown residue was obtained with a yield of 0.43, 0.57, and 0.51% *w*/*w* from IG2019, IG2021, and RM2019, respectively.

### 3.4. LC-PDA-ESI-MS Analysis

#### 3.4.1. Sample Preparation

Stock solutions of purified samples of the alkaloids **1**–**6** were prepared by dissolving 0.5 mg mL^−1^ of each compound in dichloromethane (DCM) and storing at 4 °C. Equal aliquots of each alkaloid stock solution were mixed, diluted (1:100, *v*/*v*) with the mobile phase (A:B, 95:5, *v*/*v*, vide infra), and the resulting working solution was used to optimize the chromatographic separation and the ESI source parameters. Diluted solutions (1:100, *v*/*v*, with A:B, 95:5, *v*/*v*) of individual alkaloids were then analysed in triplicate by the HPLC-PDA-ESI-MS in SIR mode optimized method (described below) for their unambiguous identification. The same individual alkaloid solutions were used for the alkaloids’ ESI mass spectral characterization by direct infusion experiments (described below).

Stock solutions of the samples IG2019, IG2021, and RM2019 were prepared by dissolving 1 mg mL^−1^ of dry extracts in DCM and storing them at 4 °C. Equal aliquots of each sample were then diluted (1:100, *v*/*v*) with the mobile phase (A:B, 95:5, *v*/*v*), filtered (0.22 μm), and injected (20 μL) in triplicate for the analysis by the HPLC-PDA-ESI-MS in the SIR mode optimized method.

#### 3.4.2. HPLC-PDA-ESI-MS in SIR Mode Method

All samples were analysed with a Waters system composed of a 1525μ HPLC (Milford, MA, USA), a 996 PDA detector, and a Quattro Micro Tandem MS/MS with a Waters ESI source (Micromass, Manchester, UK), by using a Supelco Ascentis^®^ Express C18 (15 cm × 2.1 mm) 2.7 μm analytical column. Milli-Q water/formic acid 5 mM (A) and acetonitrile/formic acid 5 mM (B) were used as the mobile phase, flowing at 0.20 mL/min. The chromatographic separation was optimized as follows: 0 min, 5% B; 0–50 min, 80% B; 50–55 min, 80% B; 55–56 min, 5% B; and 56–76 min, 5% B, to equilibrate the column. The PDA detector recorded one UV–vis spectrum per second in the range of 200–800 nm, with a resolution of 1.2 nm.

The matrix in the whole was explored by acquiring mass spectral data in full scan mode within the mass range 80–1000 Da, in positive ionization (ES+) and in negative ionization (ES-); the positive ionization ES+ was chosen to optimize the selected ion recording (SIR) method, used to identify selected alkaloids by acquiring the *m*/*z* monoisotopic values corresponding to the protonated ions [M + H^+^]^+^ in separate channels. The ESI source parameters were optimized as follows: capillary voltage = 3.0 kV; cone voltage = 25 V; source temperature = 120 °C; desolvation temperature = 350 °C; cone gas flow = 30 L/h; desolvation gas flow = 550 L/h; and dwell cell = 0.200 s. MassLynx Software 4.1 v (Data Handling System for Windows, Micromass, UK) was used for instrument control, data acquisition, and data handling.

#### 3.4.3. DI-ESI-MS/MS Experiments

Experiments were carried out by infusing the samples directly into the ESI source through an external syringe, with a flow rate of 5 µL/min. ES+ mass spectral data were acquired for 2 min in the appropriate mass range, with a cone voltage of 25 V, ionization source temperature of 100 °C, desolvation gas temperature of 150 °C, cone gas flow of 30 L/h, and desolvation gas flow 400 L/h. Alkaloid fragmentation patterns were obtained by selecting the precursor ion, using argon as collision gas and optimized collision energy (CE) in the range of 15–38 eV, and acquiring spectra for 2 min in the appropriate mass range. All acquisitions were carried out in duplicate.

### 3.5. NMR Analysis

Selected samples of *V. sardoa* non-polar extracts, namely RM2019, IG2019, and IG2021, were dried and resuspended in 700 µL of deuterated chloroform containing hexamethyldisiloxane at a concentration of 2 mM as both chemical shift and concentration internal standard. ^1^H-NMR spectra were acquired as previously reported [21].

### 3.6. Cell Culture

Cell culture SUP-B15, a B lymphoblast cell line isolated from the marrow of an 8-year-old, male patient with acute lymphoblastic leukaemia, and HDF fibroblast cells (human dermal fibroblast) were both obtained from ATCC, Manassas, VA, USA. SUP-B15 cells were grown in McCoy’s 5A modified medium (ATCC) while fibroblast cells were grown in Dulbecco’s modified Eagle’s medium (DMEM, Euroclone, Pero, Italy) both with the addition of 10% foetal bovine serum (HyClone™ Fetal Bovine Serum, USA origin, Characterized), 100 units/mL penicillin, and 100 units/mL streptomycin at 37 °C in a humidified atmosphere with 5% CO_2_.

#### MTT Assay

SUP-B15 cells were plated at a density of 1.2 × 10^4^ cells in a 96-well cell-culture-treated, U-shaped-bottom microplate (Costar) while fibroblast cells were seeded for 24 h in a 96-well plate (flat-bottom) (Corning^®^, Corning, NY, USA) with a density of 1.0 × 10^4^ cells/well. Both cell lines were resuspended in 90 μL of culture medium. Then, the cells were exposed (10 µL) to increased concentrations of the extracts of IG2019, IG2021, and RM2019 (1 µg/mL, 5 µg/mL, 10 µg/mL, and 15 µg/mL) in a cell culture medium for 24, 48, and 72 h. After the incubation period, 20 µL of 5 mg/mL MTT solution (Sigma, Deisenhofen, Germany) was added to each well for 2 h at 37 °C in 5% CO_2_ atmosphere; cells were dissolved with 100 μL of MTT solvent (4 mM HCl, 0.1% NP40 in isopropanol). The absorbance was read on a microtiter spectrophotometric plate reader at 570 nm, in the same experiments, and standardized to 100% with respect to control cells. The results obtained were calculated as absorbance. All data were obtained in triplicate and from three independent experiments. Vincristine and vinblastine, used as reference drugs, were purchased from Merck (Milan, Italy).

## 4. Conclusions

Three extracts (IG2019, IG2021, and RM2019) of *V. sardoa* of plants collected from different locations were prepared following an acid–water extraction methodology. The phytochemical analyses carried out by NMR and LC-MS revealed that IG2019, IG2021, and RM2019 contain indole alkaloids identified as 14,15-didehydroaspidofractinine (1), venalstonine (2) *N*(1)-formyl-14,15-didehydro-12-hydroxyaspidofractinine (3), conoflorine (4), *N*(1)-methyl-14,15-didehydroaspidofractinine (5), and *N*(1)-methyl-14,15-didehydro-12-methoxyaspidofractinine (6). All the extracts were tested on lymphoblast cell line SUP-B15 at different times (24, 48, and 72 h) and concentrations (1 µg/mL, 5 µg/mL, 10 µg/mL, and 15 µg/mL). All the extracts were able to induce a reduction in cell proliferative capacity without showing a cytotoxic effect on normal cells. To the best of our knowledge, this is the first study investigating the biological activity of *V. sardoa*. Our promising preliminary results encourage us to further investigate the mechanism of action of *V. sardoa* extracts on leukemic cells.

## Figures and Tables

**Table 1:** Alkaloids **1**–**6** identified in IG2019, IG2021, and RM2019 extracts by HPLC-PDA-ESI-MS analysis in SIR mode, in positive ionization (ES+). t_R_: retention time (min), given as medium value ± standard deviation from triplicate analysis; M: molecular mass (Da); [M + H^+^]^+^: monoisotopic mass/charge value of the protonated molecular ion of alkaloids (*m*/*z*); the main fragments for each compound; CE: collision energy.

Alkaloid	t_R_ (min)	M (Da)	[M + H^+^]^+^ (*m*/*z*)	Principal Fragments (*m*/*z*)	CE (eV)
**1**	6.77 ± 0.13	306	307	262, 144	30
**2**	7.04 ± 0.12	336	337	320, 144, 260	33
**3**	7.41 ± 0.17	322	323	278, 160	30
**4**	9.29 ± 0.21	296	297	154, 144, 279	28
**5**	11.74 ± 0.21	292	293	158, 173, 144	35
**6**	12.22 ± 0.15	322	323	188, 203, 174, 144	35

**Table 2:** Quantified alkaloids in *V. sardoa* extracts. Values are expressed as mg/100 g of dried extract.

	RM2019	IG2019	IG2021
Alkaloids **1**, **2**, **5**	177.96 ± 8.89	38.7 ± 1.94	273.85 ± 13.65
Alkaloid **4**	167.22 ± 8.36	57.66 ± 2.88	135.09 ± 6.76
Alkaloids **3**, **6**	177.21 ± 8.86	113.89 ± 5.69	99.93 ± 4.95

## Data Availability

Data sharing not applicable.

## Supplementary Materials

The following supporting information can be downloaded at: https://www.mdpi.com/article/10.3390/molecules28155639/s1, Figure S1: Alkaloid **1**: structure, molecular mass, *m*/*z* monoisotopic value of the protonated ion [M + H^+^]^+^, mass spectrum (bottom), fragmentation spectrum at 30 eV (CE) (top), tentatively assigned principal fragments. Figure S2: Alkaloid **2**: structure, molecular mass, *m/z* monoisotopic value of the protonated ion [M + H^+^]^+^, mass spectrum (bottom), fragmentation spectrum at 33 eV (CE) (top), tentatively assigned principal fragments. Figure S3: Alkaloid **3**: structure, molecular mass, *m/z* monoisotopic value of the protonated ion [M + H^+^]^+^, mass spectrum (bottom), fragmentation spectrum at 30 eV (CE) (top), tentatively assigned principal fragments. Figure S4: Alkaloid **4**: structure, molecular mass, *m/z* monoisotopic value of the protonated ion [M + H^+^]^+^, mass spectrum (bottom), fragmentation spectrum at 28 eV (CE) (top), tentatively assigned principal fragments. Figure S5: Alkaloid **5**: structure, molecular mass, *m/z* monoisotopic value of the protonated ion [M + H^+^]^+^, mass spectrum (bottom), fragmentation spectrum at 35 eV (CE) (top), tentatively assigned principal fragments. Figure S6: Alkaloid **6**: structure, molecular mass, *m/z* monoisotopic value of the protonated ion [M + H^+^]^+^, mass spectrum (bottom), fragmentation spectrum at 35 eV (CE) (top), tentatively assigned principal fragments. Figure S7: Total Ion Chromatogram (TIC) and Selected Ion Recording (SIR) chromatograms of the extracts of *V. sardoa*, RM2019 sample. (a) TIC; (b) SIR chromatogram obtained for the selected *m/z* value 307, corresponding to the protonated ion [M + H]^+^ of alkaloid **1**, t_R_ = 6.77 min; (c) SIR chromatogram obtained for the selected *m/z* value 337, corresponding to the protonated ion [M + H]^+^ of alkaloid **2**, t_R_ = 7.04 min; (d) SIR chromatogram obtained for the selected *m/z* value 323, corresponding to the isobaric protonated ions [M + H]^+^ of alkaloid **3**, t_R_ = 7.41 min, and alkaloid **6**, t_R_ = 12.22 min; (e) SIR chromatogram obtained for the selected *m/z* value 297, corresponding to the protonated ion [M + H]^+^ of alkaloid **4**, t_R_ = 9.29 min; (f) SIR chromatogram obtained for the selected *m/z* value 293, corresponding to the protonated ion [M + H]^+^ of alkaloid **5**, t_R_ = 11.74 min. Figure S8: ^1^H-NMR (600 MHz, CDCl_3_) spectrum of *V. sardoa* extract. Figure S9: Cell viability analysis in fibroblast cell treated with IG2019, IG2021, RM2019 for 24, 48 and 72 h. All experiments were performed in triplicate and all data were expressed at mean ± SD values. Figure S10: Cell viability analysis in SUP-B15 cells treated with Vincristine and Vinblastine for 24, 48 and 72 h. All experiments were performed in triplicate and all data were expressed at mean ± SD values.

## Sample Availability

Samples of the plant material and extracts are available from the authors.
