# Supplementary material for: Phytochemical Analysis and In Vitro Antileukemic Activity of Alkaloid-Enriched Extracts from Vinca sardoa (Stearn) Pignatti
Source: Molecules. 2023 Jul 25;28(15):5639. doi: 10.3390/molecules28155639 (PMC10419533; doi:10.3390/molecules28155639)
Supplement: Supplementary file 1 [file molecules-28-05639-s001.zip › molecules-2472037-supplementary.pdf]

molecules

# Supplementary Materials

# Phytochemical Analysis and *In Vitro* Antileukemic Activity of Alkaloid-Enriched Extracts from *Vinca sardoa* (Stearn) Pignatti

**Daniela De Vita [1,*], Claudio Frezza [1], Fabio Sciubba [1,2], Chiara Toniolo [1], Camilla Badiali [1], Rita Petrucci [3], Martina Bortolami [3], Paola Di Matteo [3], Daniele Rocco [3], Annarita Stringaro [4], Marisa Colone [4], Andrea Maxia [5], Maria Teresa Petrucci [6], Mauro Serafini [1] and Sebastiano Foddai [1]**

[1] Department of Environmental Biology, "Sapienza" University of Rome, Piazzale Aldo Moro 5, 00185 Rome, Italy; claudio.frezza@uniroma1.it (C.F.); fabio.sciubba@uniroma1.it (F.S.); chiara.toniolo@uniroma1.it (C.T.); camilla.badiali@uniroma1.it (C.B.); mauro.serafini@uniroma1.it (M.S.); sebastiano.foddai@uniroma1.it (S.F.)
[2] NMR-Based Metabolomics Laboratory, "Sapienza" University of Rome, Piazzale Aldo Moro 5, 00185 Rome, Italy
[3] Department of Basic and Applied Sciences for Engineering, "Sapienza" University of Rome, Via Castro Laurenziano 7, 00161 Rome, Italy; rita.petrucci@uniroma1.it (R.P.); martina.bortolami@uniroma1.it (M.B.); p.dimatteo@uniroma1.it (P.D.M.); daniele.rocco@uniroma1.it (D.R.)
[4] National Center for Drug Research and Evaluation, Italian National Institute of Health, Viale Regina Elena 299, 00161 Rome, Italy; annarita.stringaro@iss.it (A.S.); marisa.colone@iss.it (M.C.)
[5] Laboratory of Economic and Pharmaceutical Botany, Department of Life and Environmental Sciences, Università degli Studi di Cagliari, Viale S. Ignazio da Laconi 13, 09123 Cagliari, Italy; a.maxia@unica.it
[6] Department of Cellular Biotechnologies and Hematology, "Sapienza" University of Rome, Via Benevento 6, 00161 Rome, Italy; petrucci@bce.uniroma1.it
* Correspondence: daniela.devita@uniroma1.it; Tel.: +39-06-4991-2788

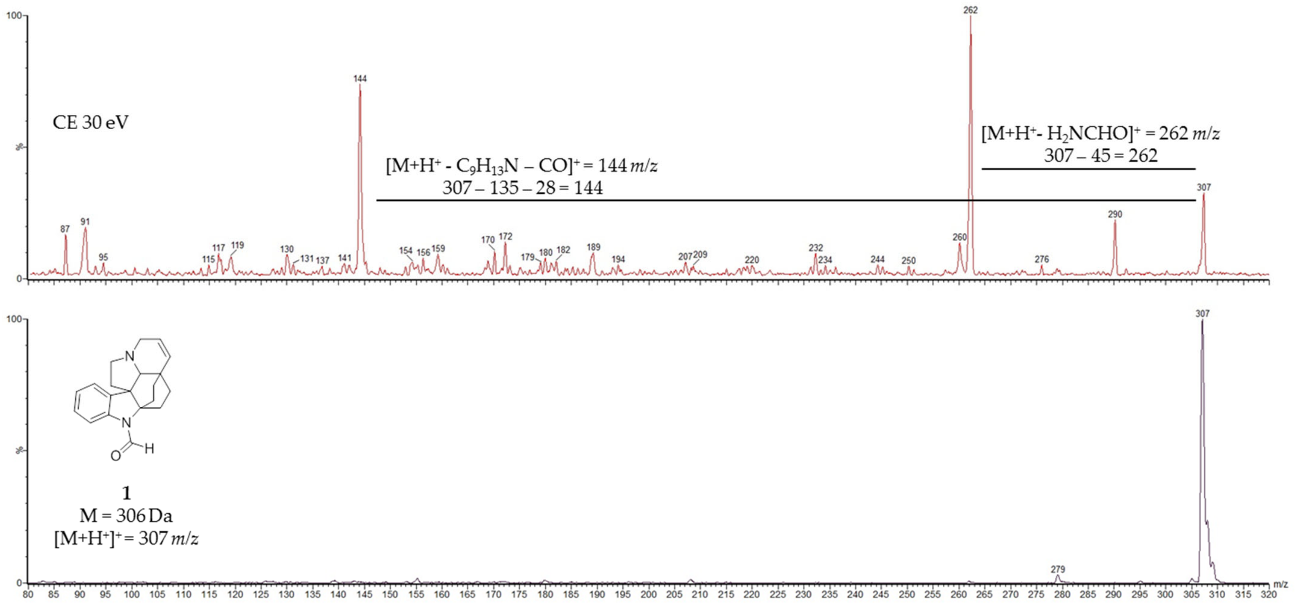


**Figure S1.** Alkaloid **1**: structure, molecular mass, *m/z* monoisotopic value of the protonated ion $[M + H^+]^+$, mass spectrum (bottom), fragmentation spectrum at 30 eV (CE) (top), tentatively assigned principal fragments.

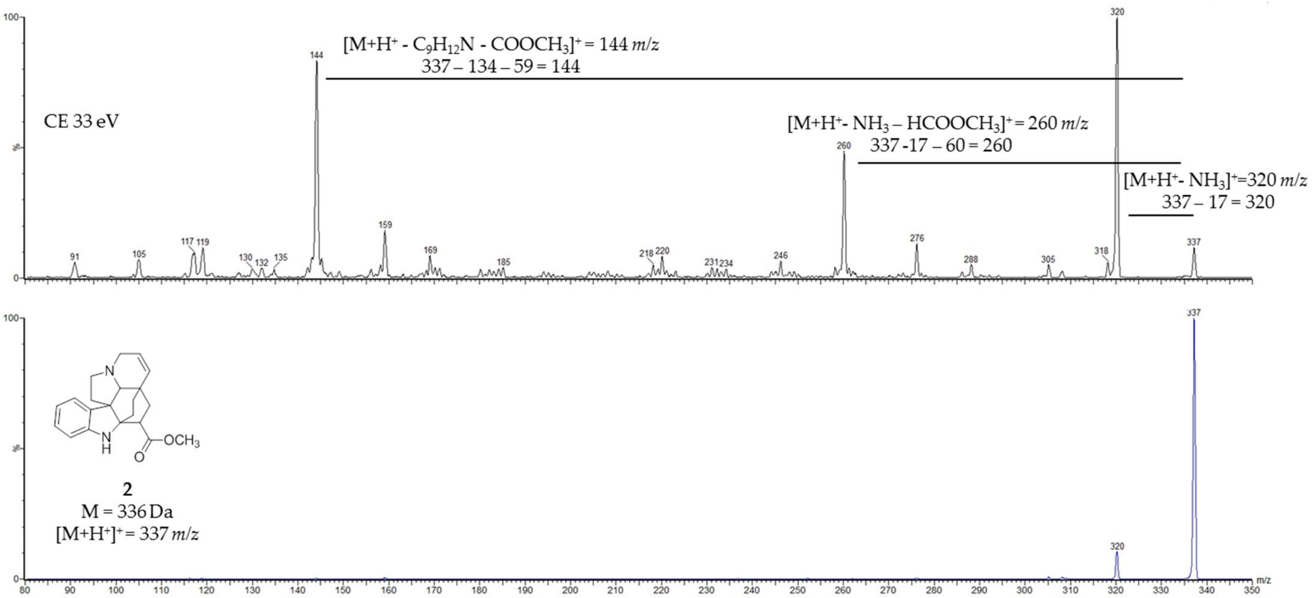


**Figure S2.** Alkaloid **2**: structure, molecular mass, *m/z* monoisotopic value of the protonated ion $[M + H^+]^+$, mass spectrum (bottom), fragmentation spectrum at 33 eV (CE) (top), tentatively assigned principal fragments.

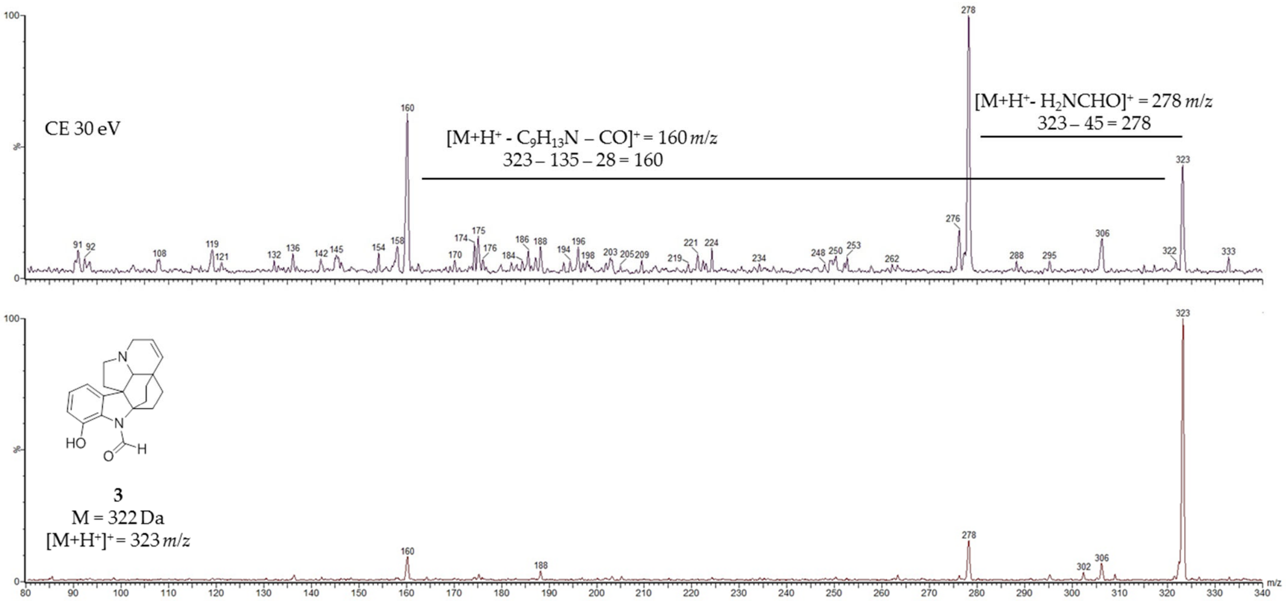


**Figure S3.** Alkaloid **3**: structure, molecular mass, *m*/*z* monoisotopic value of the protonated ion [M + H+]+, mass spectrum (bottom), fragmentation spectrum at 30 eV (CE) (top), tentatively assigned principal fragments.

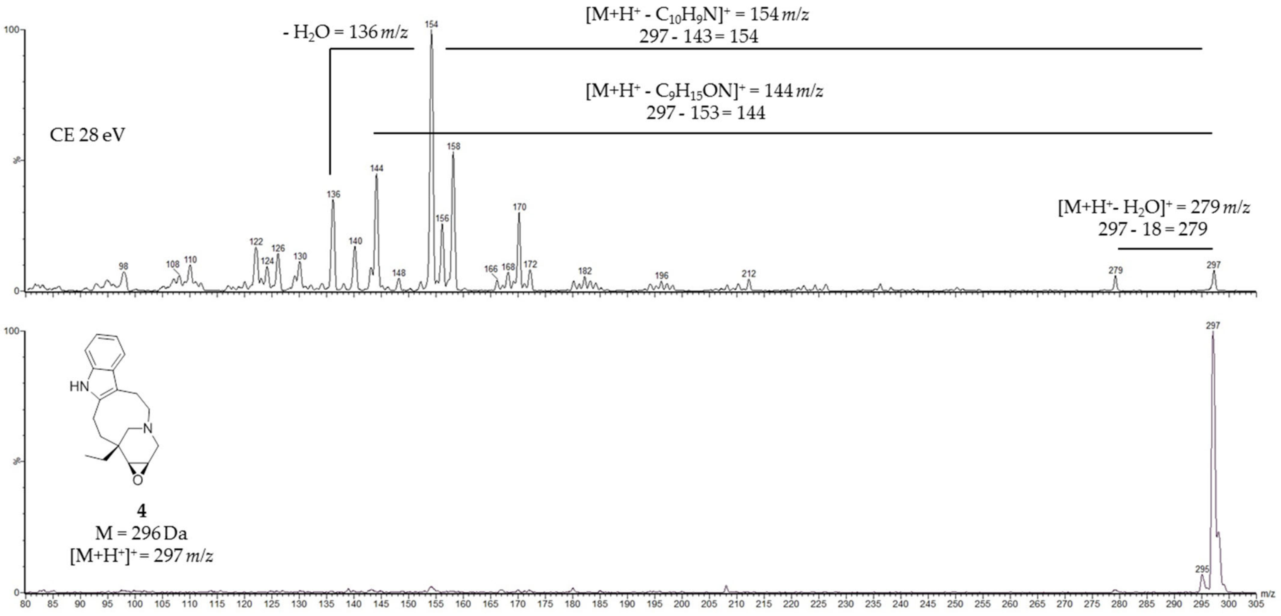


**Figure S4.** Alkaloid **4**: structure, molecular mass, *m*/*z* monoisotopic value of the protonated ion [M + H+]+, mass spectrum (bottom), fragmentation spectrum at 28 eV (CE) (top), tentatively assigned principal fragments.

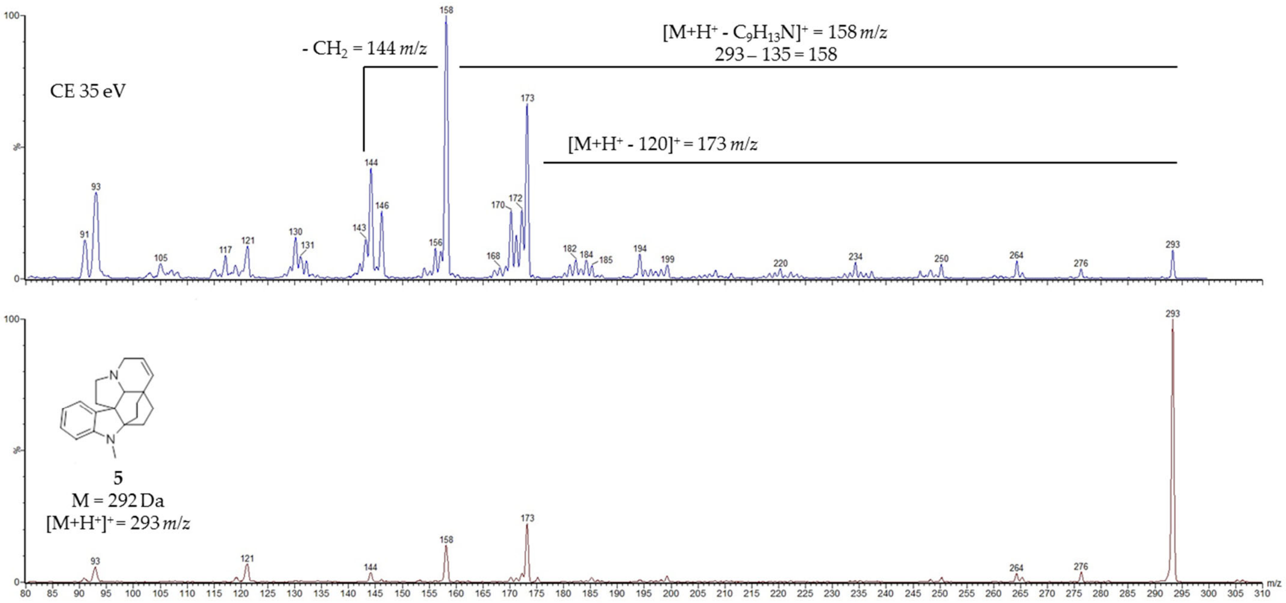


**Figure S5.** Alkaloid **5**: structure, molecular mass, *m/z* monoisotopic value of the protonated ion [M + H⁺]⁺, mass spectrum (bottom), fragmentation spectrum at 35 eV (CE) (top), tentatively assigned principal fragments.

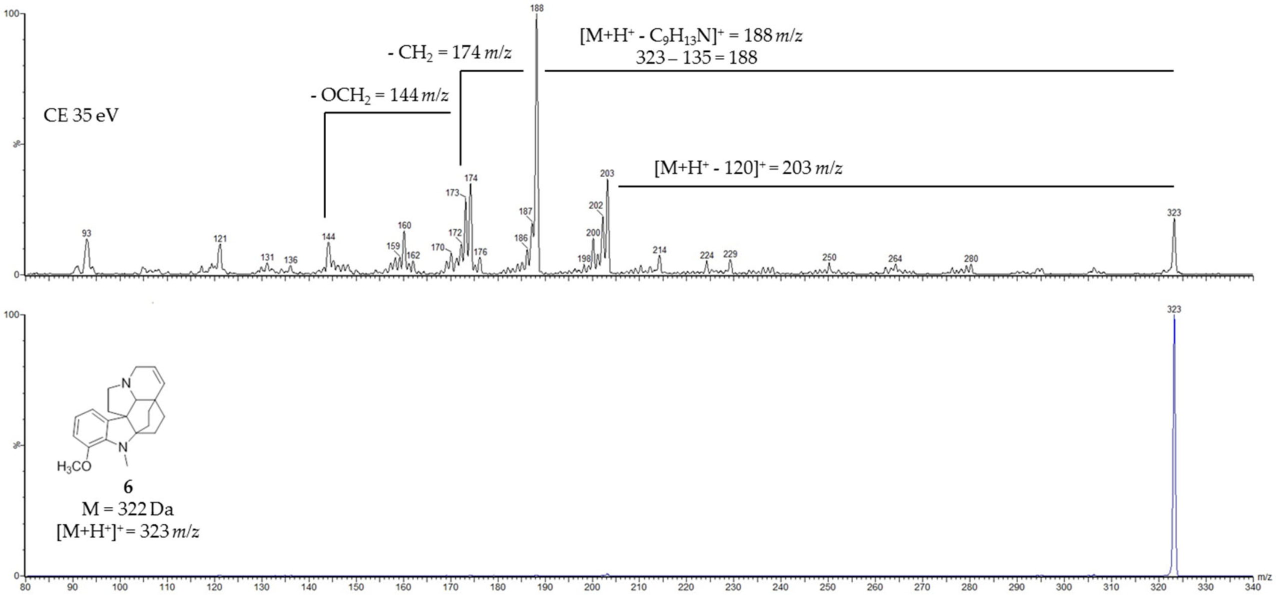


**Figure S6.** Alkaloid **6**: structure, molecular mass, *m/z* monoisotopic value of the protonated ion [M + H⁺]⁺, mass spectrum (bottom), fragmentation spectrum at 35 eV (CE) (top), tentatively assigned principal fragments.

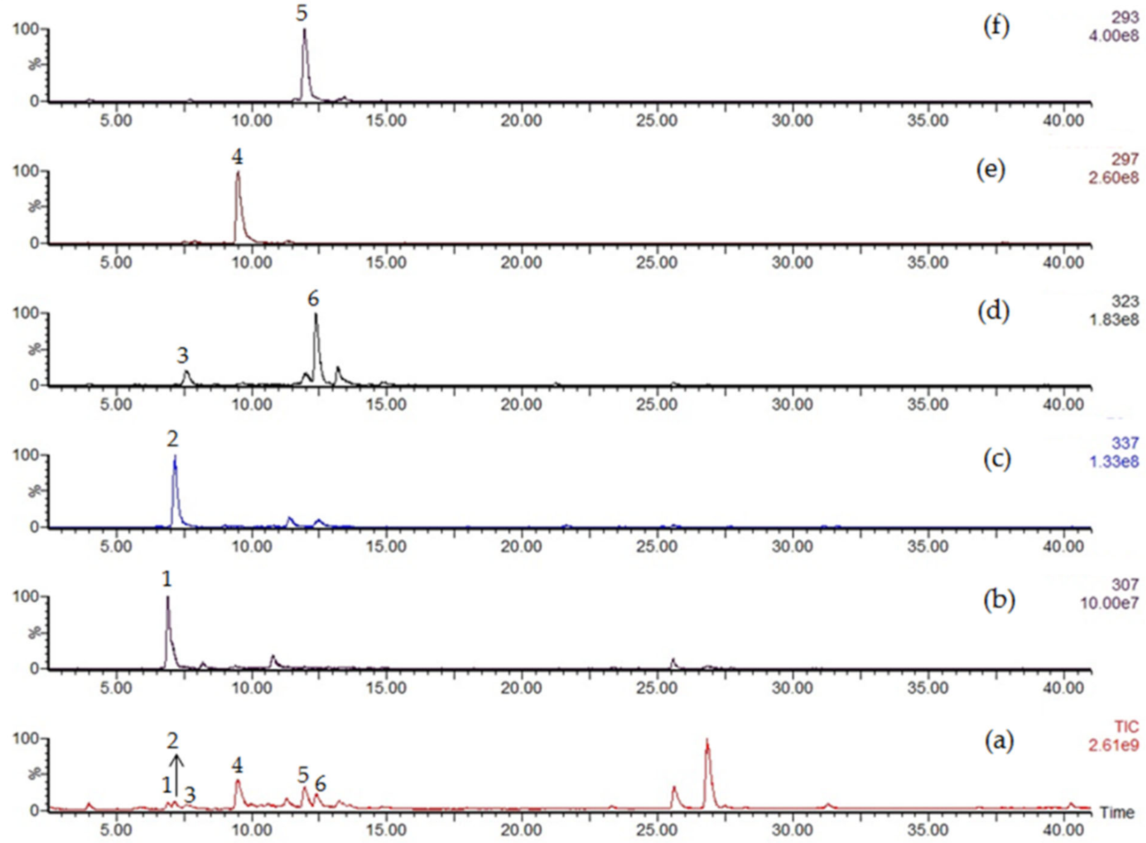


**Figure S7.** Total Ion Chromatogram (TIC) and Selected Ion Recording (SIR) chromatograms of the extracts of *V. sardoa*, RM2019 sample. (a) TIC; (b) SIR chromatogram obtained for the selected *m*/*z* value 307, corresponding to the protonated ion $[M + H]^+$ of alkaloid **1**, $t_R$ = 6.77 min; (c) SIR chromatogram obtained for the selected *m*/*z* value 337, corresponding to the protonated ion $[M + H]^+$ of alkaloid **2**, $t_R$ = 7.04 min; (d) SIR chromatogram obtained for the selected *m*/*z* value 323, corresponding to the isobaric protonated ions $[M + H]^+$ of alkaloid **3**, $t_R$ = 7.41 min, and alkaloid **6**, $t_R$ = 12.22 min; (e) SIR chromatogram obtained for the selected *m*/*z* value 297, corresponding to the protonated ion $[M + H]^+$ of alkaloid **4**, $t_R$ = 9.29 min; (f) SIR chromatogram obtained for the selected *m*/*z* value 293, corresponding to the protonated ion $[M + H]^+$ of alkaloid **5**, $t_R$ = 11.74 min.

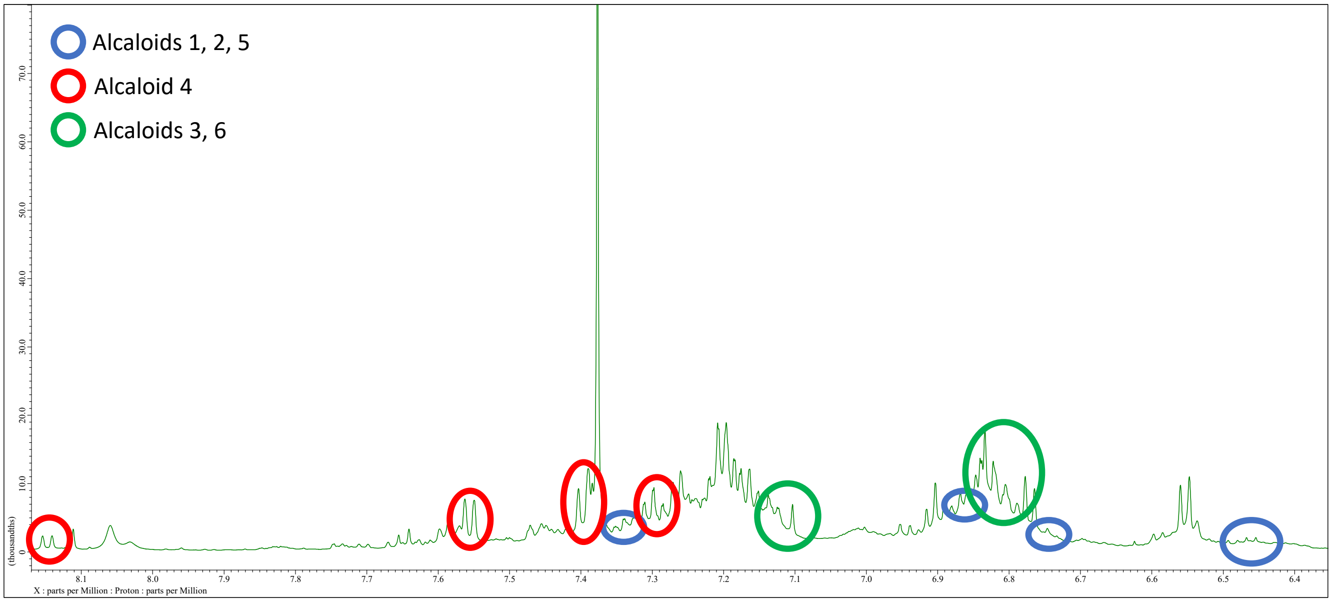


**Figure S8.** $^1$H-NMR (600 MHz, $CDCl_3$) spectrum of *V. sardoa* extract.

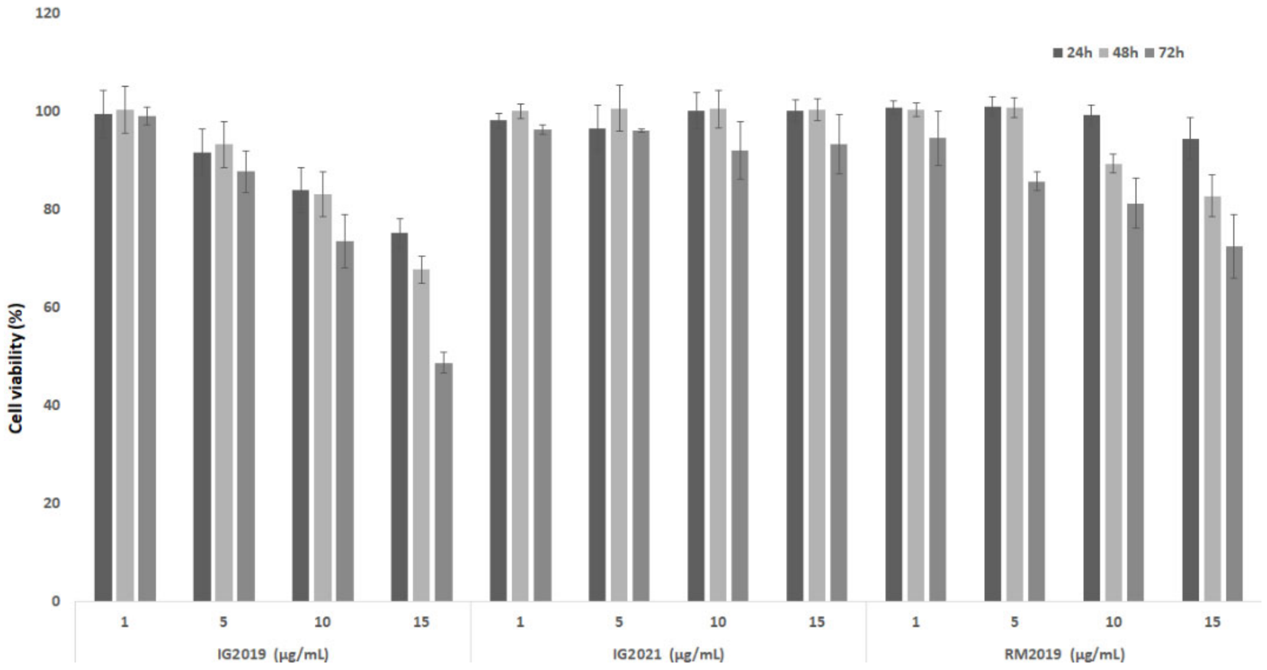


**Figure S9.** Cell viability analysis in fibroblast cell treated with IG2019, IG2021, RM2019 for 24, 48 and 72 h. All experiments were performed in triplicate and all data were expressed at mean ± SD values.

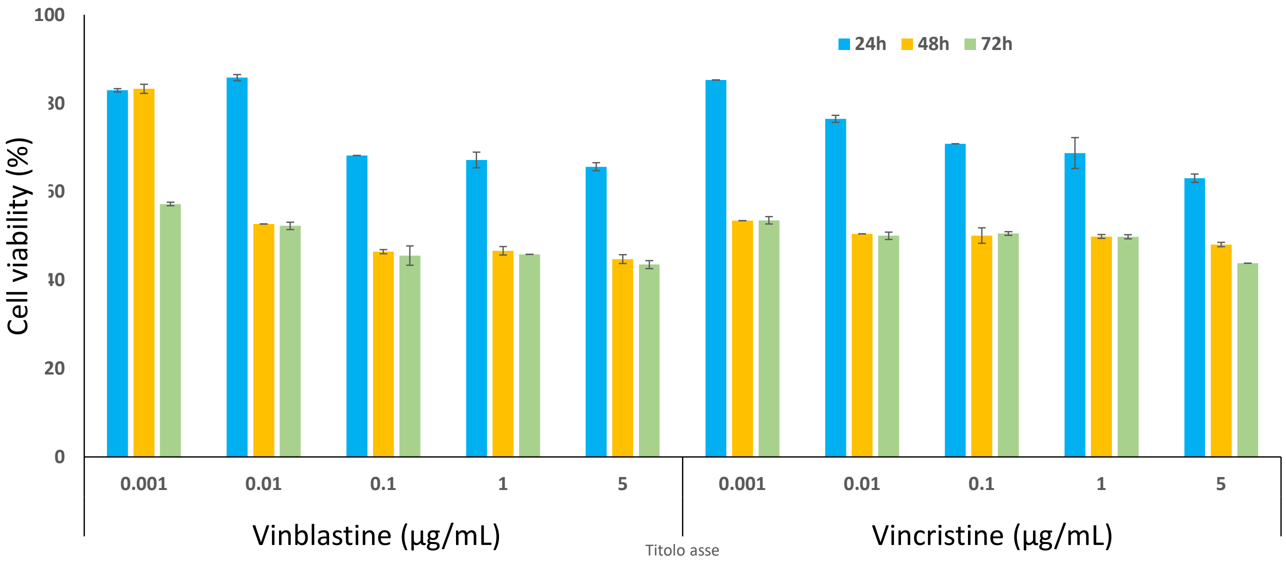


**Figure S10.** Cell viability analysis in SUP-B15 cells treated with Vincristine and Vinblastine for 24, 48 and 72 h. All experiments were performed in triplicate and all data were expressed at mean ± SD values.
